# Cranberry: Chemical Composition, Antioxidant Activity and Impact on Human Health: Overview

**DOI:** 10.3390/molecules27051503

**Published:** 2022-02-23

**Authors:** Boris V. Nemzer, Fadwa Al-Taher, Alexander Yashin, Igor Revelsky, Yakov Yashin

**Affiliations:** 1 Department of Research & Development, VDF FutureCeuticals, Inc., Momence, IL 60954, USA; bnemzer@futureceuticals.com; 2 Department of Food Science and Human Nutrition, University of Illinois at Urbana-Champaign, Urbana, IL 61801, USA; 3 Chemistry Department, Lomonosov Moscow State University, 119992 Moscow, Russia; yashinchromat@yandex.ru (A.Y.); yashinchrom@mail.ru (Y.Y.); 4 International Analytical Center, Zelinsky Institute of Organic Chemistry at Russian Academy of Sciences, 119991 Moscow, Russia; i.revelsky@gmail.com

**Keywords:** cranberry, anthocyanins, antioxidant, proanthocyanidins, urinary tract infection, polyphenols

## Abstract

Cranberries are a rich source of bioactive compounds that comprise a healthy diet. Cranberry is abundant in nutritional components and many bioactive compounds that have antioxidant properties. Both American (*Vaccinium macrocarpon*) and European (*Vaccinium oxycoccus*) cranberry species are rich in polyphenols such as phenolic acids, anthocyanins and flavonoids, and is one of the few fruits that is high in proanthocyanidins, which is linked to many health benefits. The review systematizes information on the chemical composition of cranberry, its antioxidant effect, and the beneficial impact on human health and disease prevention after cranberry consumption, and in particular, its effect against urinary tract inflammation with both adults and children, cardiovascular, oncology diseases, type 2 diabetes, metabolic syndrome, obesity, tooth decay and periodontitis, Helicobacter pylori bacteria in the stomach and other diseases. Additional research needs to study cranberry proteomics profiling, polyphenols interaction and synergism with other biologically active compounds from natural ingredients and what is important in formulation of new functional foods and supplements.

## 1. Introduction

In North America, the ‘large’ cranberry is referred to as *Vaccinium macrocarpon*, and is cultivated in the northern parts of the country. It is mainly produced in New Jersey, Massachusetts, Oregon, Washington, Wisconsin and, in the Canadian provinces of British Columbia and Quebec [1,2,3]. They are also grown for trade purposes in Chile [2]. The ‘large’ cranberry is also present in the commercial farms of Europe such as in Germany, Belarus, Latvia, Lithuania and Russia [3]. 

These regions have suitable environmental conditions for cranberries to grow in including sandy soil, plenty of fresh water and a cool resting period for which they can thrive in the growing season [2]. The indigenous people of North America gave the cranberry its English name because the stem, calyx and petals resembled the neck, head and bill of a crane and berry stands for English berry, thus “craneberry” [3]. 

The ‘small’ cranberry, also called *Vaccinium oxycoccus,* is harvested in the wild and present in Ireland, the British Isles and Scandinavia, eastern and central Europe, Finland and Germany, the Balkan countries, and Siberia and Japan [2,3,4]. It is sold in the markets of the Baltic states, Finland, Poland, Ukraine, and Russia [2,3].

Cranberries grow on trailing vines in beds layered with sand, peat, gravel and clay, which are called bogs or marshes [2]. The ‘large’ cranberry is an evergreen shrub with low growing vines that produce slender wiry stems up to 2 m long and 5–20 cm in height [3,4,5]. Leaves are elliptical and grow up to 22 mm long and 9 mm wide [3]. They can also grow light pink flowers [3,4,5]. Bees pollinate them. ‘Large’ cranberry fruits can be red, dark red, or dark purple, while those that have not completely matured are a pale pink or white color, which accounts for red and white cranberry juices [3,4]. The ‘small’ cranberry grows on peat in poorly drained areas with high water with stems that can extend up to 100 cm long [3]. Leaves are ovate and may expand up to 16 mm long and 6 mm wide [3]. 

Ripening of ‘large cranberry’ cultivars begins near the end of summer to the beginning of September and continues through October in the U.S. and Canada [3,5]. The ‘small cranberries’ ripen from late August through September and can be under the snow until spring [3]. The ‘small cranberry’ has more tolerance to cold than the ‘large cranberry’ [3]. Only about 5% of cranberries produced in the U.S. are sold fresh and the remaining 95% are processed into products such as juices, sauce, and dried fruits because of their tart flavor [1,3,6,7]. Americans consume an estimated 400 million pounds of cranberries per year, 20% of them during the holidays. The U.S. per capita consumption of cranberries is 2.3 pounds, primarily as juice or juice blends [6]. In 2019, world production of cranberry was 687,534 tons with the U.S. being the primary producer (359,111 tons), followed by Canada (172,440 tons) and Chile (141,338 tons) [8]. Cranberry production in the U.S. for 2020 totaled about 7.8 million barrels, mainly by Wisconsin (4.6 million barrels) and Massachusetts (2.1 million barrels) with the least by New Jersey (0.5 million) and Oregon (0.6 million) [7]. The U.S. total area harvested was 39,300 acres [7]. 

Early European colonists discovered the benefits of cranberry as a medicine and as a food product in North America [1]. Both ‘small’ and ‘large’ cranberries have been used over several decades in North America, and some parts of Asia and Europe, to prevent or cure different diseases [3]. They are mostly known for treating or preventing urinary tract infections and maintaining the digestive system [3].

Cranberry is abundant in nutritional components and many bioactive compounds that have antioxidant properties. Both American and European cranberry species are rich in many classes of phytochemicals. These include phenolic acids, anthocyanins, flavones, flavonoids, and organic acids. Cranberry is one of the few fruits that is high in proanthocyanidins, which inhibit adherence of *Escherichia coli* to the urinary tract [2,3,5,9,10]. The content of phenolic compounds in the cranberries is influenced by aspects such as cultivar, agriculture practices, geographical area, weather conditions, ripeness, harvesting time, and storage settings [3]. The greatest quantity of total phenols is accrued at the beginning of berry ripening [3]. The cultivars grown in colder weather are characterized by higher amounts of phenolics than the same cultivars grown in a mild climate [3]. Consuming cranberries can prevent tooth decay and gum disease, inhibit urinary tract infections, reduce inflammation in the body, maintain a healthy digestion system, and decrease cholesterol levels [2,5,9,10]. This investigation summarizes recent scientific studies as to the health benefits of cranberry due to its phytochemical and antioxidant activity. This review can help promote cranberries as functional foods for consumers interested in maintaining their well-being and reducing health risks the natural way.

## 2. Chemical Constituents of Cranberry Fruit

### 2.1. Nutritionals

Raw, unsweetened American cranberries contain mainly 87% water and 12% carbohydrates, with lesser amounts of protein, fats and fiber (Table 1) [11]. Small cranberries accrue 2.1–4.9% titrable acidity with citric acid contributing 1.8–2.6%. Large cranberries accumulate less titrable acidity (1.9–2.4%) with citric acid found to be 1.88 mg/g and 6.08 mg/g for ‘Howes’ and the ‘Early Black’ cultivars, respectively [3]. Citric, malic and quinic acids were the main acids found in the large cranberry [3]. Cranberry nutritional composition may differ depending on the cultivar, climate, growing conditions, maturity/ripeness stage, time of harvest and storage conditions [3,9,10,11,12,13,14,15]. Oszmiański et al. [12] reported a total acidity range from 1.95 to 2.35 g/100 g fm for six cultivars grown in Poland [12]. These are comparable to results of Oszmiański et al. [13] who detected acidity from 2.2 to 2.3 g/100 g for three cranberry cultivars tested. Ĉesoniene et al. [15] noticed different amounts of organic acid content in 40 gentoypes (27 wild clones and 13 certified cultivars) of cranberry fruit (*Vaccinium oxycoccos*) of European origin (Estonian, Russian and Lithuanian) grown under similar agricultural conditions in Lithuania. The concentration of quinic acid was 3.81–13.3 g/kg, of malic acid was 14.1–43.3 g/kg and of citric acid was 10.8–54.3 g/kg [10,15]. 

Glucose (3.44 g), fructose (0.67 g), and sucrose (0.16 g) contribute to the simple sugars in 100 g of raw cranberries (Table 1) [11]. Large cranberries constitute 3.4 to 7.1% of monosaccharides, while small cranberries have a lesser amount of sugars, 2.2 to 6.0%. Cranberries contain mostly glucose and fructose with glucose accounting for 58.9 to 65.9% of monosaccharides. The large cranberry contains more sucrose (3.9–5.3%) than in the small cranberry (0.01–0.5%) [3]. Oszmiański et al. [12] determined glucose to be the predominant sugar in cranberry with a range of 3.36 to 4.72 mg/100 g fm in six cultivars with the total sugar content of 3.83 mg/100 g to 4.82 mg/100 g fm. Ĉesoniene et al. [15] noticed cranberry fruit had similar amounts of fructose (42.1 g/kg) and glucose (45.1 g/kg). Total sugar concentration increased with ripening for the three cultivars of *Vaccinium macrocarpon* ‘Pilgrim’ (38.4%), ‘Stevens’ (34.9%) and ‘Ben Lear’ (40.9%) [13]. Fructose was the main sugar identified in these cranberry fruit cultivars with a range of 58.9 to 68.7% of total sugar, followed by glucose ranging from 29.6 to 39.3% and sucrose ranging from 1.7 to 1.9%. The sweetness of the cranberries is due to these three monosaccharides, fructose, glucose and sucrose [13]. 

Cranberries contain a wide range of water-soluble and fat-soluble vitamins (Table 1). Antioxidants in cranberries predominantly come from a rich source of Vitamin C, Vitamin E and Vitamin K [3,11]. The small cranberry comprises 15.3 to 30% of ascorbic acid, the main active form of Vitamin C. A higher amount (47.5%) was detected in the large cranberry [3]. Of the six cultivars of cranberry fruit (*Vaccinium macrocarpon*) grown in a horticultural farm with similar conditions in a region of Poland, ‘Pilgrim’ had the highest content of Vitamin C (20.74 mg/100 g fresh matter (fm)) and ‘Red Star’ had the lowest content (10.07 mg/100 g fm) [12]. Viskelis et al. [14] determined the largest ascorbic acid content was found in ripe berries (15.8 mg/100 g). Cranberry also contains a small amount of fat, Omega-3 and Omega-6, which are important for the human diet (Table 1) [11].

Minerals encompassed 0.19 to 0.28% fresh weight (fw) of the small cranberry [3]. European cranberries (*Vaccinium oxycoccos*) and American cranberries (*Vaccinium macrocarpon*) showed similar average mineral content for the macronutrients, potassium (67.19 and 72.51 mg/100 g, respectively), calcium (12.74 and 10.19 mg/100 g, respectively), and sulfur (8.14 and 7.85 mg/100 g, respectively) [16]. Statistically significant differences (*p* < 0.05) were found for nitrogen (54.9 and 42.1 mg/100 g, respectively), phosphorus (6.12 and 8.59 mg/100 g, respectively), and magnesium (8.09 and 6.61 mg/100 g, respectively) for the cranberries. Potassium and nitrogen were the major minerals identified in these two varieties of cranberry fruits [3,16]. American cranberry contained a greater mean amount of iron (0.72 mg/100 g) than the European variety (0.31 mg/100 g). Manganese and boron (2.59 and 0.09 mg/100 g, respectively) were higher in the European cranberry fruit than the American cranberry (0.19 and 0.065 mg/100 g, respectively) [16]. This suggests cranberry fruit is a good source of minerals.

### 2.2. Biochemical Constituents

Cranberries contain chemically diverse, secondary metabolites, polyphenols that have antimicrobial and antioxidant properties [1,3]. Chemical composition of cranberries varies due to environmental conditions as well as the ripening process [3]. Cranberry cultivars attain the appropriate shape, weight, texture, color, aroma and flavor during the ripening stage. 

Both types of cranberries, the large and small, contain many phenolic compounds, such as phenolic acids, flavonoids (anthocyanins and flavonols), and tannins. The large cranberry has been recognized as an important food and healing agent because of these compounds. Flavonoids were the major compounds identified among over 150 compounds in the large cranberry [3]. Flavonoids are classified into subgroups that include anthocyanins, flavonols, and proanthocyanidins. The large cranberry was found to contain 13 anthocyanins, 16 flavonols, and 26 phenolic acids and benzoates [3]. The small cranberry includes flavonoids, such as anthocyanins, catechins, and flavones [3]. Forty-eight polyphenols (including 19 flavonols, 8 anthocyanins, 7 phenolic acids and 14 flavan-3-ol oligomers) were identified in three cranberry cultivars (‘Pilgrim’, ‘Stevens’, and ‘Ben Lear’) in a study of different maturity ripening stages in Poland [13]. The lowest amount of polyphenols was observed in the immature and semi-mature ripening stages and increased in the overripe, commercially mature cranberry. In order from highest to the lowest amounts of polyphenol classes that were detected in the cranberry fruits are flavan-3-ols (41.5–52.2%), flavonols (18.6–30.5%), anthocyanins (8.0–24.4%), and phenolic acids (5.0–12.1%) [13]. Among the three cranberry species, *Vaccinium macrocarpon* Ait., *Vaccinium* *oxycoccos* L., and *Vaccinium vitisidaea* (lingonberry grown in North America and Europe), 4624 compounds were identified with about 8000–10,000 phytochemicals found in each type [10,17]. 

#### 2.2.1. Phenolic Acids

Cranberries constitute phenolic acids that include hydroxybenzoic and hydroxycinnamic acids [3,10,18,19,20]. Cranberry has high amounts of benzoic acid and lower amounts of 2,4-dihydroxybenzoic acid, *p*-hydroxybenzoic, and *o*-hydroxybenzoic acids. The hydroxycinnamic acids in cranberry are *p*-coumaric, sinapic, caffeic, and ferulic acids [18]. The total amount of phenolic compounds in cranberry fruit cultivars depends on the cultivar and berry ripening. 

Abeywickrama et al. [19] observed *p*-coumaric acid as the most abundant compound followed by caffeic acid, ferulic acid and chlorogenic acid in a cranberry wild clone and the cultivar ‘Pilgrim’ (Table 2). Kylli et al. [20] found hydroxycinnamic acid (129.1 mg/100 g dry weight) and hydroxybenzoic acid (0.9 mg/100 g dry weight) in the *Vaccinium macrocarpon* cranberry. Kalin et al. [21] detected *p*-coumaric acid (13.0 mg/kg), *p*-hydroxybenzoic acid (55 mg/kg), kaempherol-3-O-glucoside (11.0 mg/kg), caffeic acid (5.0 mg/kg) and ellagic acid (3.0 mg/kg) as the main phenolic compounds in extracts of *Vaccinium macrocarpon*. 

Total phenolic acids ranged from the lowest concentration of 327 mg/100 g dry matter (dm) in ‘Pilgrim’ to the highest content of 649 mg/100 g dm in ‘Howes’ when six cultivars of cranberry fruit grown in Poland were tested [12]. Cranberries accrued the highest amount of total phenols at the beginning of the ripening process [3,13]. The average concentration of phenolic acids in the three cranberry cultivars during the ripening process ranged from 236.8 mg/100 g dm in ‘Ben Lear’ to 351.5 mg/100 g dm in ‘Pilgrim.’ The content of phenolic acids in ‘Ben Lear’ decreased somewhat (about 2.8%) from the immature to semi-mature phase and then increased from the semi-mature to mature phase by around 15.1% and then decreased from the mature to the commercially mature (over-ripe) phase by about 21.6%. There was a similar trend for ‘Pilgrim’ at 26.5, 0.02 and 5.9%, respectively, and ‘Stevens’ at 34.5, 2.4 and 5.3%, respectively. The main phenolic acid compounds found in the cranberry cultivars were caffeoyl hexoside and caffeoyl dihexoside [13].

#### 2.2.2. Anthocyanins

Anthocyanins are natural water-soluble pigments that give cranberries their reddish color. Anthocyanins in the small berry were found to be 6 to 10 times higher in the external layer of the berry skin than in the pulp [3,14]. 

The content of total anthocyanins ranged from 695 to 1716 mg/100 g dm for the six cultivars of *Vaccinium macrocarpon* L. grown in Poland [12]. Of one hundred and thirty-six wild cranberry fruits, *Vacciniun macrocarpon* Aiton, and two cultivars, ‘Franklin’ and ‘Bergman’, Debnath and An [22] observed the total anthocyanins content for all cranberry genotypes to differ significantly (*p* < 0.05). Variation ranged from the least amount of total anthocyanins in the wild clone cranberry NL77 of 0.13 mg/g fw to the highest amount detected in the genotype ‘Franklin’ of 2.27 mg/g fw [22]. In another study by Česonienė et al. [15], six anthocyanins were identified in the two species of cranberries, *Vaccinium macrocarpon* and *Vaccinium oxycoccos*. These are cyanidin-3-galactoside, cyanidin-3-glucoside, cyanidin-3-arabinoise, peonidin-3-galactoside, peonidin-3-glucoside and peonidin-3-arabinoside. 

Of the three cranberry fruits (*Vaccinium macrocarpon* L.), ‘Ben Lear’ consisted of the highest average level of anthocyanins (690.4 mg/100 g dm) when analyzed at the four different maturity stages of ripening and was 50.6% and 6.0% higher than ‘Pilgrim’ and ‘Stevens’, respectively. There was a sharp increase in anthocyanins content in ‘Pilgrim’, ‘Stevens’, and ‘Ben Lear’ (57.3%, 47.0% and 30.0%, respectively) during ripening from the immature stage to the commercially mature stage [13]. Viskelis et al. [14] also determined the highest content of anthocyanins in overripe cranberries. The major anthocyanins identified in the cranberry cultivars in the ripening study were cyanidin-3-O-galactoside (32.6–45.0% of total anthocyanins) and peonidin-3-O-galactoside (22.7–32.2%) [13]. Abeywickrama et al. [19] detected cyanidin-3-O-arabinoside and cyanidin-3-O-galactoside in the cranberry Canadian wild clone NL2 and ‘Pilgrim’. This shows that the anthocyanins concentration in cranberry fruits is dependent on cultivar and ripening stage [12,13,22]. 

#### 2.2.3. Flavonols

Flavonols are abundant in cranberries [12,13,18,19]. Flavonol content ranged from 643 to 1088 mg/100 g dm in the six cultivars grown in Poland [12]. Abeywickrama et al. [19] identified four major flavonols in the cranberry Canadian wild clone NL2 and ‘Pilgrim’ cultivar. These were quercetin 3-O-rhamnoside, myricetin 3-O-arabinoside, quercetin 3-O-galactoside, and myricetin 3-O-galactoside. The flavonol glycosides identified in *Vaccinium vitisidaea* cranberry by Diaconeasa et al. [23] were myricetin-galactoside, quercetin-rutinoside (rutin), myricetin-arabinoside, myricetin-glucoside, quercetin-galactoside, quercetin-acetyl-glucoside, quercetin-glucoside and quercetin-rhamnoside. Debnath and An [22] showed significant differences (*p* <0.05) in the total flavonoid content among the 136 wild clones and two cultivars ranging from 2.78 to 7.51 for the wild clones, NL51 and NL92, respectively. The cultivar ‘Franklin’ (5.81 mg/g fw) had more total flavonoids than ‘Bergman’ (4.06 mg/g fw) [22]. About 15 of the clones from three provinces had higher amounts of total flavonoids than the two cultivars [22]. 

Flavonol concentrations show a slight increase in cranberries during ripening [3,13]. ‘Pilgrim’ contained the greatest amount of flavonols (average of 1201.6 mg/100 g dm) when tested at the different maturity stages of ripening and was 36.1% and 18.7% higher than for ‘Ben Lear’ and ‘Stevens’, respectively. Flavonols increased in the cranberry cultivars from the immature to the commercially mature stage by 25%, 9% and 1% in ‘Pilgrim’, ‘Stevens’, and ‘Ben Lear’, respectively [13]. The major flavonols noticed in these cranberry fruit cultivars were quercetin-3-O-galactoside (31.3–38.4% of total flavonols), myricetin-3-O-galactoside (20.4–29.0%) and quercetin-3-O-pentoside (10.1 to 11.8%) [13]. 

#### 2.2.4. Flavonoids (Flavan-3-ols and Proanthocyanidins)

Flavonoids are important in plant defense and are strong antioxidants. They also exhibit antibacterial, antiviral, anticarcinogenic, and anti-inflammatory activities [3]. Flavonoids identified in six cultivars of cranberries ranged from the lowest amount of 860 mg/100 g dm in ‘Red Star’ to the highest amount of 1283 mg/100 g dm in ‘Howes’ (Table 2). Polymeric procyanidins ranged from a low of 651 mg/100 g dw in ‘Ben Lear’ to the highest amount of 1109 mg/100 g dw in ‘Red Star’ [12]. Kylli et al. [20] noted that proanthocyanidins comprised 71% of the total phenolic content in cranberries. ‘Ben Lear’ was observed to contain an average of 1733.1 mg/100 g dm flavan-3-ols and 1958.1 mg/100 g dm in ‘Pilgrim’ in the *Vaccinium macrocarpon* L. cultivars at the different ripening stages [12]. The concentrations of proanthocyanidins varied in the cultivars ‘Pilgrim’, ‘Stevens’, and ‘Ben Lear,’ and were higher in the early stage of ripening but then decreased quickly by around 9.4, 15.2 and 19.0%, respectively, when the cranberry fruits were over ripe [13]. 

Jungfer et al. [24] noticed that proanthocyanins A-type trimers varied in the three species of cranberries, the large American cranberry, *Vaccinium* *macrocarpon* Ait., the small European cranberry, *Vaccinium oxycoccus*, and the lingonberry, *Vaccinium vitis-idaea* L. Only two A-type trimers were detected in *Vaccinium oxycoccus. Vaccinium vitis-idaea* showed the greatest variation with a pattern similar to that of *Vaccinium macrocarpon* [24]. B-type trimers were found in all berries. *Vaccinium macrocarpon* and *Vaccinium oxycoccus* exhibited higher amounts of A-type than B-type trimers and dimers [24]. The amount of procyanidin A2 in the *Vaccinium macrocarpon* varieties ranged from 4.10 to 5.49 mg/100 g of fresh berries. For *Vaccinium vitis-idaea*, the concentration of procyanidin A2 was 2.11 mg/100 g fw for the European berries and 7.98 mg/100 g fw for the Chinese berries. The lowest concentration of procyanidin A2 was observed in the *Vaccinium oxycoccus* berries at a range of 0.13–0.21 mg/100 g fw. *Vaccinium vitis-idaea* had the highest amount of A-type dimer, followed by *Vaccinium macrocarpon* and the least by *Vaccinium oxycoccus* [24]. Abeywickrama et al. [19] determined that the A-type proanthocyanins were more abundant than the B-type proanthocyanins in wild clone NL2, whereas the proanthocyanidin trimer B and trimer A were predominant in ‘Pilgrim’. The study also determined that the cranberries wild clone NL2 had a somewhat higher amount of proanthocyanidins (~1175 µg/g) than the cultivar ‘Pilgrim’ (~1047 µg/g) [19] (Table 2). 

Cranberry is distinct from most foods and other berry fruits in that it is rich in the A-type proanthocyanidins, which inhibit the in vitro adhesion of *Esherichia coli* bacteria to uroepithelial cells to prevent urinary tract infections [18]. Table 2 summarizes all the concentrations of proanthocyanidins A-type dimers, trimers, tetramers and B-type dimers and trimers detected in the cranberry fruit in various studies. 

The flavan-3-ols, catechin and epicatechin were observed in all berry samples [12,20,24]. (−) Epicatechin is the major constituent of proanthocyanidins, while (+) catechin and (epi) gallocatechins exist in small amounts [18]. The catechin to epicatechin ratios in the cranberry species were found to be different [24]. *Vaccinium vitis-idaea* had the highest total content of 15.48 mg/100 g fw for the European variety and 17.68 mg/100 g fw for the Canadian variety, while the *Vaccinium macrocarpon* varieties had a range of 2.80–5.05 mg/100 g fw and the lowest concentration was in the *Vaccinium oxycoccus* varieties at a range of 0.55–1.94 mg/100 g fw [24]. Borges et al. [25] detected (−)-epicatechin and proanthocyanidin dimers in *Vaccinium oxycoccus* cranberries, but not monomers, which contributes to the antioxidant capacity of the berries. European cranberry contained more epicatechins and less A-type dimers than lingonberry [20]. 

All varieties of the *Vaccinium macrocarpon* had higher amounts of epicatechin (2.45–4.46 mg/100 g fw) than catechin (0.33–0.61 mg/100 g fw), while the ratio of epicatechin to catechin in *Vaccinium oxycoccus* (1:1) was lower. This suggests that different ratios of the flavan-3-ols may depend on the origin of the berries [24]. Additionally, the concentrations of the flavan-3-ols, (+) catechin and (−) epicatechin increased in ‘Pilgrim’ (25%) during ripening from the immature stage to the commercially mature stage but decreased in ‘Stevens’ and ‘Ben Lear’ (2.0 and 36.8%, respectively) [13].

#### 2.2.5. Polyphenols

The amount of total phenolic compounds for the five American cranberry cultivars *Vaccinium macrocarpon* (Pilgrim, Ben Lear, Stevens, Early Richard and Bergman) ranged from 192.1 mg/100 g fm (‘Pilgrim’) to 374.2 mg/100 g fm (‘Ben Lear’) as compared to the European wild-grown cranberry *Vaccinium oxycoccus* at 288.5 mg/100 g [26]. The highest anthocyanin content was found in ‘Early Richard’ at 77.1 mg/100 g fm and the lowest content in ‘Ben Lear’ at 52.1 mg/100 g fm [26]. This was lower for the wild cranberry at 43.4 mg/100 g fm [26]. Tikuma et al. [27] observed the *Vaccinium macrocarpon* Ait. cultivar ‘Early Black’ contained the highest amount of anthocyanins and phenolic (105 and 441 mg 100 g^−1^, respectively) than ‘Stevens’, ‘Bergman’, ‘Pilgrim’, ‘Septembra’ and the wild cranberry *Vaccinium oxycoccus* L. The results showed there are statistically significant differences (*p* < 0.05) between the biochemical constituents of the cranberry cultivars and the species [27].

#### 2.2.6. Triterpenoids

Cranberry fruits also contain triterpenoids. Two of the major triterpenoids, ursolic acid and its isomer, oleanic or oleanolic acid are found in the wax of the skin of the cranberry fruit and are mostly responsible for anti- inflammatory, antitumor and anticancer activities [12,28]. Ursolic acid is present in *Vaccinium oxycoccus* and protects against oxidative damage and lipid oxidation [10] and has strong anti-inflammatory effects [10,18].

Wu et al. [28] identified ursolic acid, oleanolic acid, *β*-sitosterol, and stigmasterol in *Vaccinium macrocarpon* cranberry extracts. *β*-sitosterol and stigmasterol showed a total of 107.83 mg/g. The cranberry extract also contained ursolic acid and oleanic acid at concentrations of 372.97 mg/g and 79.16 mg/g, respectively. Oszmiański et al. [12] detected betulinic acid, oleanic acid, and ursolic acid in the fruit. The concentrations of the acids differed between the cultivars and ranged from 37–50% for ursolic acid, followed by 28–35% for oleanolic acid, and 19–28% for betulinic acid [12]. Higher amounts of total triterpenoids were found in ‘Franklin’ (3671 mg/kg dm) and lower ones in ‘Pilgrim’ (2892 mg/kg dm) [12]. 

The average content of triterpenoids for ‘Pilgrim’, ‘Stevens’, and ‘Ben Lear’ was 2528 mg/kg dm, 2736 mg/kg dm, and 3201 mg/kg dm, respectively, during the maturing stage of the three cranberry cultivars [13]. The concentrations of the triterpenoids increased by 9.0% in ‘Pilgrim’, 24.1% in ‘Stevens’ and 22.6% in ‘Ben Lear’ during ripening. The ursolic acid content in the cultivars ranged from 22.7–32.2% of total triterpenoids and the amount increased as the fruit ripened [13].

Table 2 summarizes all the phytochemicals detected in cranberry fruit with the various analytical techniques listed.

## 3. Antioxidant Activity

Cranberry fruit is an important source of antioxidants, such as polyphenols (flavonoids, phenolic acids, anthocyanins, tannins), ascorbic acid, and triterpene compounds [9,10]. They scavenge free radicals, unpaired electrons in their outer orbit and may remove reactive oxygen species that oxidize biological matter [9,10,17]. Oxidative stress, extreme amounts of free oxygen radicals in the biological fluids in the human body can cause many diseases [9,10,28]. Antioxidant compounds can prevent or reduce oxidative damage to cell structure. Antioxidant activity is influenced by cultivar, genotype, growing season, ripening, processing and storage of cranberry fruit [9]. Their role is critical to preventing the development of chronic diseases such as cardiovascular diseases, aging, diabetes, inflammation, cancer, etc. [9,10,22,29,30]. 

Borowska et al. [26] compared the antioxidant properties of wild cranberry fruit (*Vaccinium* oxycoccus) and five American cranberry cultivars and observed a statistically significant difference (*p* < 0.05) between the wild cranberry fruit and the cultivars. The wild cranberry fruit possessed greater antioxidant activity (AOC) [26]. This study observed the highest scavenging capacity by DPPH radicals to be within the range of 33.87–68.83 µmol/g of fresh mass. ‘Stevens’ had the highest antioxidant capacity followed by ‘Pilgrim’ when assessed using all three types of free radicals (DPPH, OH and ABTS) [26].

The total content of antioxidants in cranberry was found to be 270 mg/100 g when measured by an amperometric method [29]. Borges et al. [25] found the total antioxidant activity by FRAP assay in *Vaccinium oxycoccus* cranberry to be 18.6 µmol of Fe^2+^/g, whereas Diaconeasa et al. [23] noted 19.6 µmol Fe^2+^/g. The greatest amount of antioxidant activity detected by ABTS, DPPH, and FRAP assays in the different cultivars of *Vaccinium macrocarpon* was in ‘Franklin’ at 264, 320, and 139 µmol TE/g dm, respectively [12]. The lowest antioxidant activity observed using the ABTS, DPPH, and FRAP assays was in ‘Pilgrim’ at 189, 214, and 93.25 µmol TE/g dm, respectively. The antioxidant activity (ABTS, DPPH and FRAP) correlated positively with the total polyphenolic compounds (R^2^ = 0.508, 0.584, 0.591), anthocyanins (R^2^ = 0.675, 0.602, 0.614), and flavonols (R^2^ = 0.646, 0.724, 0.728) in cranberry fruit. The antioxidant activity also correlated with the triterpenoids (R^2^ = 0.852, 0.737, 0.736). Ursolic acid showed the strongest correlation with the ABTS, DPPH and FRAP assays ((R^2^ = 0.833, 0.559 and 0.553, respectively) [12]. Abeywickrama et al. [19] and Viskes et al. [14] concluded that the use of various methods for the determination of antioxidant activity showed that the polyphenols extracted from the berries have good radical scavenging activity and are antioxidant compounds.

A significant difference was observed (*p* < 0.05) for the antioxidant activity with the DPPH assay among 136 wild cranberry genotypes and two cultivars tested by Debnath and An [22]. Results ranged from 1.17 to 2.53 mg/g for the wild clones and 2.27 mg/g for the cultivar ‘Franklin’ and 1.63 mg/g for ‘Bergman’. High antioxidant activities were observed despite the differences due to genotypes [22]. The antioxidant capacity increased with ripening from the immature to the commercially mature stage for ‘Pilgrim’ (ABTS-21.7%; FRAP-22.1%), ‘Stevens’ (ABTS-24.9%; FRAP-21.9%), and ‘Ben Lear’(ABTS-31.9%; FRAP-28.1%) [13].

Ascorbic acid is known for its high antioxidant activity since it neutralizes free radicals and other reactive oxygen species which cause tissue damage and diseases [9]. In a study by Brown et al. [17] of the three species of cranberry, *Vaccinium macrocarpon* Ait., *Vaccinium oxycoccos* L., and *Vaccinium vitisidaea*, antioxidant activity showed a negative correlation with the anthocyanin content and a positive correlation with Vitamin C. Borges et al. [25] noticed that Vitamin C has the highest antioxidant capacity (AOC) of 22.6% and (−)-epitecatechin is the major phenolic compound detected at 1121 nmol/g and with peonidin-3-O-galactoside contributing only 14% of the overall AOC. Anthocyanins are the second major group with up to 725 nmol/g (39% of total AOC) of cranberries. A total of 456 nmol/g of flavonols were present (10% of the overall AOC) [25].

## 4. Effect of Cranberry Consumption on Human Health

European colonists who arrived in North America immediately recognized cranberries’ healing powers, such as poultice for wounds and cure for blood poisoning [1]. Cranberries best-known benefits have been to treat urinary tract infections, which is due to proanthocyanidins (PACs). These tannins prevent *Escherichia coli (E. coli)* bacteria from attaching to cells in the urinary tract and causing infection [2]. Today, more health benefits have been shown due to the phytochemicals, anthocyanins, PACs, and flavonols, found in cranberries. They reduce certain infections, promote a healthy heart, decrease inflammation associated with chronic disease and aging and support digestive health [2,31]. Cranberries also contain phytochemicals that act as antioxidants, which reduce oxidative damage to cells that can lead to cancer, heart disease, and other degenerative diseases [2,31].

In a study of the cranberry effects on human health, since 1984, several articles and reviews have been published. The most common issues on the cranberry effect on human health are investigated in reviews [18,31,32,33]. Cranberries have an anti-bacterial effect and in various forms (juice, concentrated powders, capsules and tablets) have been traditionally used to treat cystitis and urinary tract infections (UTIs) [34,35,36,37,38,39,40,41,42,43,44,45,46,47,48,49,50,51,52,53,54,55,56,57,58,59,60,61,62]. A study noted that the consumption of cranberry juice reduced the number of UTIs by 39% in women [34]. This is mainly due to the PACs content in cranberry [35], especially proanthocyanidin A [36]. Several studies have confirmed the positive effect of cranberry on urinary tract inflammation, not only for adults [34,35,36,37,38,39,40,41,42,43,44,45,46,47] but also for children [48,49,50]. It was found that PACs contained in cranberries prevent adherence of *E. coli* to uroepithelial cells in the urinary tract [44,45,56].

In clinical studies, products containing cranberry were found to prevent recurrent UTIs in young women and middle-aged women [41,57,58]. Cranberry juice prevented recurrence of UTIs in children with a 65% reduction shown in the study by Afshar et al. [49] and 43% in the Salo et al. [50] study. On the other hand, cranberry products were found not to significantly reduce recurrence of UTI [51,52]. Among otherwise healthy college women with an acute UTI, those drinking 8 oz of 27% cranberry juice twice daily did not experience a decrease in the 6-month incidence of a second UTI [52]. Although several studies have shown consuming cranberries had a protective effect against UTI [18,34,55], other studies have not seen positive effects [53,54].

Men receiving radiation for prostate cancer usually have a side effect of acute radiation cystitis, inflammation of the bladder. There is no effective treatment for preventing or treating radiation cystitis. In a pilot study, Hamilton et al. [59] determined that the incidence of cystitis was lower in men (65%) when taking cranberry capsules (containing 72 mg PACs) compared with those that took placebo capsules (90%). This study concluded that it may be beneficial for men receiving radiation therapy for prostate cancer to take cranberry capsules instead of antibiotics or ant-inflammatory drugs [59].

Antibiotics are also used to treat women with chronic cystitis. However, there has been adverse effects and increased risk of resistance with antibiotics. Studies show that cranberry can be used as an alternative [56,60]. Proanthocyanidins in the cranberries can remove *E. coli* adhesion to the urothelium [56]. The occurrence of acute cystitis decreased when treated by cranberries [56,60,61,62]. A study found that cranberry, D-mannose, a gelling complex, and the two microorganisms *Lactobacillus plantarum* and *Lactobacillus paracasei* LPCO9 significantly improved the uncomfortable symptoms associated with acute cystitis in women [61]. It was suggested that an alternative to antibiotics in the treatment of cystitis and recurrent UTIs are cranberry products [62]. However, there is no conclusive evidence that antibiotics can be replaced completely with cranberry [60,62].

Cranberry has a strong anti-oxidant property due to the presence of its rich polyphenol content such as flavonoids, proanthocyanidin dimers and oligomers, which prevents oxidative stress, the precursor to many chronic diseases [63]. Consuming cranberry juice increases the plasma antioxidant capacity while significantly reducing lipid oxidation in women with health problems [64,65].

Cranberry helps treat cardiovascular diseases, improving the lipid profile, minimizing the likelihood of atherosclerosis by decreasing low density lipoproteins, reducing blood pressure and increasing high density lipoproteins (good) and preventing metabolic syndrome [66,67,68,69,70,71,72,73]. Cranberry consumption lowers the risk of type 2 diabetes [74].

Cranberry is effective against all inflammatory processes, and now it is known that even cardiovascular and oncology diseases lead up to inflammatory responses. Cranberry can be used to prevent stomach ulcers by suppressing the activity of the *Helicobacter pylori* bacterium in the human stomach [75,76,77,78,79,80]. It is known that this bacterium can lead to gastritis, ulcer and stomach cancer. Cranberry is active against cancer [81,82,83]. Some phytochemicals in cranberry fruit affect cancer-related processes. PACs and flavonoids in cranberries may limit processes involved in tumor invasion and metastasis. *Vaccinium oxycoccos* fruits can suppress the spread of breast cancer cells, which may bring about apoptosis and GI phase arrest [82]. Cranberry extracts inhibit the growth of breast, bladder, prostrate, lung and other tumors [82,83].

Cranberry consumption helps against rheumatoid arthritis with women [84]. Cranberry and its products inhibit the development of tooth decay and periodontal diseases [85,86,87,88]. Other benefits of cranberry include prevention of the following diseases: obesity [73], infectious diseases [89], and kidney disease [90]. Cranberry also improves gut microbiota [91].

Cranberries have been shown to have a high antiviral effect with a positive effect shown between anti-influenza viral activity and total polyphenol content, which indicates high amounts of polyphenols are an important factor in the antiviral effect of berries [92,93].

Cranberry also exhibited microbial activity. It slowed the growth of human pathogenic bacteria such as *E. coli*, Salmonella typhimurium, Enteroccocus faecalis, Listeria monocytogenes, Staphylococcus and Bacillus subtilis [94,95,96,97].

Table 3 summarizes all the diseases that cranberry consumption can prevent.

## 5. Conclusions

This review summarized available data on cranberry phytochemicals characterization and their impact on human health. Cranberries represent a rich source of phenolic acids and flavonoids that have been linked to various health benefits. Cranberry fruit phytonutrients include anthocyanins, phenolic acids, flavonols, flavan-3-ols, proanthocyanidins, triterpenoids and their antioxidant activities. It has been shown that consumption of cranberry offers a reliable protection from and prevention of many chronic diseases. In general, cranberry fruit has cardioprotective, anti-carcinogenic, anti-diabetic, anti-inflammatory, antipyretic, antiseptic, antibacterial, antiviral, and other effects. All this information will potentially add to an already high level of interest toward cranberry cultivars and should certainly be incorporated as an integral part of healthy, nutritious eating, and at formulation of new functional food ingredients and dietary supplements.

An increase in demand for functional foods has led to development of food products with added protein. Additional representation of cranberry with full screening of proteomics characterization is needed. This information should be helpful in describing the mechanism of biological activity of cranberry constituents in the human body. It is also important to investigate the interaction between cranberry polyphenols with different proteomics and polysaccharides during and after processing. This information can be used for optimization of the processing method and formulation of high-protein cranberry juice products so that the protein digestibility and antioxidant activity is increased after digestion [98].

Polyphenols naturally bind to proteins to form insoluble, stable colloidal protein-polyphenol particles, which when used as food ingredients provide more health benefits to consumers from additional bioactives [99]. Strauch and Lila [99] examined the effect of protein processing on the physiochemical properties of the pea protein-cranberry polyphenol system and found that chemical differences between proteins affected polyphenol binding and influenced digestibility. It was demonstrated that solubility was affected by both the process of forming particles and the protein-cranberry polyphenol binding [99]. Since it has been determined that the functional properties of the protein-cranberry polyphenol particles are impacted by the properties of the protein isolate raw material, more studies need to be performed on the selection of protein isolate starting material for the desirable functional food [99].

Characterization of extractable polyphenols from cranberries have been extensively assessed. However, investigations on residuals of total polyphenols, non-extractable polyphenols have been limited [100]. More characterization of non-extractable polyphenols of cranberries used as functional foods is needed and an understanding of the mechanisms of the protective anti-inflammatory and anticancer properties and high antioxidant activity they possess.

It is also important to investigate the synergism of cranberry phenolics with other natural ingredients for different biological activities. Vattem et al. [101] studied the interaction of cranberry polyphenols with two other ingredients and noted that antimutagenic effectiveness of cranberry increased when they were added. More studies similar to this need to be performed. The summary presented in this review should be useful at formulation of new functional products.

## Figures and Tables

**Table 1:** Cranberries, raw, Nutritional value per 100 g.

Name	Amount	Name	Amount
Water	87 g	Vitamin E	1.3 mg
Energy	46 kcal	Vitamin K	5 µg
Carbohydrates	12 g	Vitamin A, as retinol	3 µg
Sugars	4.3 g	Vitamin A, IU	63 IU
Dietary fiber	3.6 g	Calcium	8 mg
Fat	0.1 g	Iron	0.23 mg
Protein	0.5 g	Magnesium	6 mg
Thiamine (B_1_)	0.012 mg	Manganese	0.27 mg
Riboflavin (B_2_)	0.02 mg	Phosphorus	11 mg
Niacin (B_3_)	0.101 mg	Potassium	80 mg
Pantothenic acid (B_5_)	0.295 mg	Sodium	2 mg
Vitamin B_6_	0.057 mg	Zinc	0.09 mg
Folate (B_9_)	1 µg	Copper	0.06 mg
Vitamin C	14 mg	Selenium	0.1 µg

Reference: [11].

**Table 2:** Phytonutrients in different cranberry fruit cultivars.

Name	Polyphenol Content and Triterpenoids	Analytical Method	Reference
**Anthocyanins**			
Delfinidyn derivatives	31.27–43.87 mg/100 g dm	LC/MS Q-TOF and UPLC-PDA-FL	[12]
Delfinidyn-3-O-glucoside	1.1–1.8 mg/100 g dm	LC/MS Q-TOF and UPLC-PDA-FL	[13]
Cyanidin derivatives	442–967 mg/100 g dm	LC/MS Q-TOF and UPLC-PDA-FL	[12]
Cyanidin-3-O-galactoside	119.9–180.0 mg/100 g dm 20.5% 19.3%	LC/MS Q-TOF and UPLC-PDA-FL HPLC-UV/MS HPLC-PDA	[13] [14] [15]
Cyanidin-3-O-glucoside	5.5–7.3 mg/100 g dm	LC/MS Q-TOF and UPLC-PDA-FL	[13]
	2.3%	HPLC-UV/MS	[14]
	2.8%	HPLC-PDA	[15]
Cyanidin-3-O-arabinoside	64.5–95.6 mg/100 g dm	LC/MS Q-TOF and UPLC-PDA-FL	[13]
	19%	HPLC-UV/MS	[14]
	20.2%	HPLC-PDA	[15]
Peonidin-3-O-galactoside	131.3–310.3 mg/100 g dm	LC/MS Q-TOF and UPLC-PDA-FL	[13]
	32.7%	HPLC-UV/MS	[14]
	29.6%	HPLC-PDA	[15]
Peonidin-3-O-arabinoside	42.9–95.2 mg/100 g dm	LC/MS Q-TOF and UPLC-PDA-FL	[13]
	6.7%	HPLC-UV/MS	[14]
	19.8%	HPLC-PDA	[15]
Peonidin derivatives	192–666 mg/100 g dm	LC/MS Q-TOF and UPLC-PDA-FL	[12]
Malvidin derivatives	29.85–58.85 mg/100 g dm	LC/MS Q-TOF and UPLC-PDA-FL	[12]
Malvidin-3-O-arabinoside	1.4–1.9 mg/100 g dm	LC/MS Q-TOF and UPLC-PDA-FL	[13]
Total anthocyanins	695–1716 mg/100 g dm	LC/MS Q-TOF and UPLC-PDA-FL	[12]
	3.60–152.2 mg/100 g dm	HPLC-UV/MS	[14]
	12.2–227.8 mg/kg dm	HPLC-PDA	[15]
	0.13–2.27 mg/g fw	pH differential method	[22]
**Phenolic acid**			
p-Coumaric acid	2–245 µg/g dw	HPLC/ESI-MS/MS	[19]
p-Coumaroyl hexose	8.6–13.9 mg/100 g dm	LC/MS Q-TOF and UPLC-PDA-FL	[13]
p-Coumaroyl hexose isomer	3.6–50.0 mg/100 g dm	LC/MS Q-TOF and UPLC-PDA-FL	[13]
p-Coumaroyl derivatives	210–451 mg/100 g dm	LC/MS Q-TOF and UPLC-PDA-FL	[12]
Chlorogenic acid	72.00–129.62 mg/100 g dm	LC/MS Q-TOF and UPLC-PDA-FL	[12]
	26.6–79.2 mg/100 g dm	LC/MS Q-TOF and UPLC-PDA-FL	[13]
	6–47 µg/g dw	HPLC/ESI-MS/MS	[19]
Caffeic acid	5–123 µg/g dw	HPLC/ESI-MS/MS	[19]
Caffeoyl hexoside	92.7–190.2 mg/100 g dm	LC/MS Q-TOF and UPLC-PDA-FL	[13]
Caffeoyl hexoside isomer	10.9–17.5 mg/100 g dm	LC/MS Q-TOF and UPLC-PDA-FL	[13]
Caffeoyl and derivatives	39.93–68.28 mg/100 g dm	LC/MS Q-TOF and UPLC-PDA-FL	[12]
Ferulic acid	4–39 µg/g dw	HPLC/ESI-MS/MS	[19]
Total phenolic acid	327–649 mg/100 g dm	LC/MS Q-TOF and UPLC-PDA-FL	[12]
**Flavonols**			
Myricetin-3-O-galactoside	156.5–348.4 mg/100 g dm	LC/MS Q-TOF and UPLC-PDA-FL	[13]
	2 µg/g dw	HPLC/ESI-MS/MS	[19]
	4.61 mg/100 g fw	HPLC-PDA and HPLC-ESI/MS	[23]
Myricetin-3-O-glucoside	1.8–6.6 mg/100 g dm	LC/MS Q-TOF and UPLC-PDA-FL	[13]
	8.68 mg/100 g fw	HPLC-PDA and HPLCESI/MS	[23]
Myricetin-3-O-pentoside	6.3–55.6 mg/100 g dm	LC/MS Q-TOF and UPLC-PDA-FL	[13]
Myricetin-3-O-glucoronide	19.0–38.5 mg/100 g dm	LC/MS Q-TOF and UPLC-PDA-FL	[13]
Myricetin-arabinoside	8–273 µg/g dw	HPLC/ESI-MS/MS	[19]
	5.68 mg/100 g fw	HPLC-PDA and HPLC-ESI/MS	[23]
Sinapoyl derivatives	4.36–5.82 mg/100 g dm	LC/MS Q-TOF and UPLC-PDA-FL	[12]
Myricetin derivatives	496–926 mg/100 g dm	LC/MS Q-TOF and UPLC-PDA-FL	[12]
Quercetin-3-O-galactoside	294.6–375.8 mg/100 g dm	LC/MS Q-TOF and UPLC-PDA-FL	[13]
	54–126 µg/g dw	HPLC/ESI-MS/MS	[19]
	12.02 mg/100 g fw	HPLC-PDA and HPLC-ESI/MS	[23]
Quercetin-3-O-pentoside	21.2–122.9 mg/100 g dm	LC/MS Q-TOF and UPLC-PDA-FL	[13]
Quercetin-3-O-glucoside	4.8–11.5 mg/100 g	LC/MS Q-TOF and UPLC-PDA-FL	[13]
	14.25 mg/100 g fw	HPLC-PDA and HPLC-ESI/MS	[23]
Quercetin-p-conmaroyl-hexoside	1.3–13.3 mg/100 g dm	LC/MS Q-TOF and UPLC-PDA-FL	[13]
Quercetin-3-O-rhamnoside	6.2–13.3 mg/100 g dm	LC/MS Q-TOF and UPLC-PDA-FL	[13]
	343 µg/g dm	HPLC/ESI-MS/MS	[19]
	7.32 mg/100 g fw	HPLC-PDA and HPLC-ESI/MS	[23]
Quercetin-rutinoside	12.0 mg/100 g fw	HPLC-PDA and HPLC-ESI/MS	[23]
Quercetin-acetyl-glucoside	13.58 mg/100 g fw	HPLC-PDA and HPLC-ESI/MS	[23]
Quercetin derivatives	107–225 mg/100 g dm	LC/MS Q-TOF and UPLC-PDA-FL	[12]
Methoxyquercetin hexoside	1.7–25.7 mg/100 g dm	LC/MS Q-TOF and UPLC-PDA-FL	[13]
Methoxyquercetin pentoside	3.4–61.0 mg/100 g dm	LC/MS Q-TOF and UPLC-PDA-FL	[13]
Methoxyquercetin derivatives	33.31–43.04 mg/100 g dm	LC/MS Q-TOF and UPLC-PDA-FL	[12]
Total flavonols	643–1088 mg/100 g dm	LC/MS Q-TOF and UPLC-PDA-FL	[12]
**Flavan-3-ols and proanthocyanidins**			
(+)-Catechin	2.79–7.53 mg/100 g dm	LC/MS Q-TOF and UPLC-PDA-FL	[12]
	19.6–24.5 mg/100 g dm	LC/MS Q-TOF and UPLC-PDA-FL	[13]
	0.33–13.01 mg/100 g fw	UHPLC-UV-MS	[24]
(−)-Epicatechin	27.46–56.84 mg/100 g dm 47.5–60.8 mg/100 g dm	LC/MS Q-TOF and UPLC-PDA-FL	[12]
	0.22–10.68 mg/100 g fw	LC/MS Q-TOF and UPLC-PDA-FL	[13]
		UHPLC-UV-MS	[24]
A-type PA-dimer	16.94–32.07 mg/100 g dm 14.5–108.7 mg/100 g dm	LC/MS Q-TOF and UPLC-PDA-FL	[12]
	0.22–9.75 mg/100 g fw	LC/MS Q-TOF and UPLC-PDA-FL	[13]
		UHPLC-UV-MS	[24]
A-type PA-trimer	27.82–76.94 mg/100 g dm	LC/MS Q-TOF and UPLC-PDA-FL	[12]
	15.1–53.8 mg/100 g dm	LC/MS Q-TOF and UPLC-PDA-FL	[13]
	0.14–10.01 mg/100 g fw	UHPLC-UV-MS	[24]
A-type PA-tetramer	41.51–65.61 mg/100 g dm	LC/MS Q-TOF and UPLC-PDA-FL	[12]
	18.2–40.1 mg/100 g dm	LC/MS Q-TOF and UPLC-PDA-FL	[13]
B-type PA-dimer	12.62–36.75 mg/100 g dm	LC/MS Q-TOF and UPLC-PDA-FL	[12]
	5.3–203 mg/100 g dm	LC/MS Q-TOF and UPLC-PDA-FL	[13]
	0.56–27.50 mg/100 g fw	UHPLC-UV-MS	[24]
B-type PA-trimer	0.04–2.93 mg/100 g fw	UHPLC-UV-MS	[24]
Polymeric proanthocyanidins	651–1109 mg/100 g dm	LC/MS Q-TOF and UPLC-PDA-FL	[12]
Sinapyl hexose	2.0–3.3 mg/100 g dm	LC/MS Q-TOF and UPLC-PDA-FL	[13]
Total flavan-3-ols and proanthocyanidins	860–1283 mg/100 g dm	LC/MS Q-TOF and UPLC-PDA-FL	[12]
Sum Phenolic compounds	3428–3936.4 mg/100 g dm	LC/MS Q-TOF and UPLC-PDA-FL	[13]
Total polyphenols	192.1–3742 mg/100 g fm	UV/Vis spectrophotometer	[26]
**Triterpenoids**			
Ursolic acid	1044–1714 mg/kg dm	LC/MS Q-TOF and UPLC-PDA-FL	[12]
	2466.1–2850.6 mg/kg dm	LC/MS Q-TOF and UPLC-PDA-FL	[13]
	372.97 mg/g fw	HPLC-DAD	[28]
Oleanolic acid	894–1137 mg/100 g dm	LC/MS Q-TOF and UPLC-PDA-FL	[12]
	16.2–25.6 mg/kg dm	LC/MS Q-TOF and UPLC-PDA-FL	[13]
	79.16 mg/g fw	HPLC-DAD	[28]
Betulinic acid	635–824 mg/kg dm;	LC/MS Q-TOF and UPLC-PDA-FL	[12]
	36.3–100.1 mg/kg dm	LC/MS Q-TOF and UPLC-PDA-FL	[13]
Sum Triterpenoids	2892–3671 mg/kg dm	LC/MS Q-TOF and UPLC-PDA-FL	[12]
	2528.0–3201.5 mg/kg dm	LC/MS Q-TOF and UPLC-PDA-FL	[13]
Total Sterols (β-sitosterol and stigmasterol)	107.83 mg/g fw	HPLC-DAD	[28]

Note 1: dm—dry matter; dw—dry weight; fm—fresh matter; fw—fresh weight. Note 2: Terminology for Analytical methods: LC/MS Q-TOF—Liquid Chromatography/Mass Spectrometry Quadrupole Time-of-Flight. UPLC-PDA-FL—Ultra-performance liquid chromatography-photodiode array-fluorescence. UHPLC UV/MS—Ultra-high performance ultra-violet mass spectrometer. HPLC-UV/MS—High-performance liquid chromatography-ultra-violet mass spectrometer. HPLC-PDA—High-performance liquid chromatography/photodiode array. HPLC/ESI-MS/MS—High-performance liquid chromatography/electrospray ionization tandem mass spectrometry. HPLC-DAD—High-performance liquid chromatography-diode array detector.

**Table 3:** Prevention of disease with cranberry consumption and proposed mechanisms.

Disease Name	Proposed Mechanism	References
Urinary tract inflammation	A-type procyanidins in cranberry demonstrate anti-adhesive activity against *E. coli* to the uroepithelial cells preventing progression of UTIs	[34,35,36,37,38,39,40,41,42,43,44,45,46,47,48,49,50,51,52,53,54,55]
Cystitis	A-type procyanidins in cranberry prevent adhesion of *E. coli* to the bladder epithelial cells preventing progression of UTIs	[56,57,58,59,60,61,62]
Oxidative stress	Polyphenols in cranberry alleviate intestinal oxidative stress and inflammation while improving mitochondrial dysfunction by quenching reactive oxygen species.	[63,64,65]
Cardiovascular	Polyphenols in cranberry may reduce the risk of cardiovascular disease by increasing the LDL resistance to oxidation, hindering platelet accumulation, decreasing blood pressure.	[66,67,68,69,70,71,72]
Obesity	Lyophilized cranberries reduced fat accumulation during preadipocyte differentiation by decreasing the number of receptors on the surface of target cells of the mRNA level of adipocyte fatty acid-binding protein (aP2), lipoprotein lipase (LPL), fatty acid synthase (FAS), hormone sensitive lipase (HSL) and perilipin 1 (PLIN1). Therefore, cranberries are effective in preventing the production of adipose tissue.	[73]
Type 2 diabetes	Cranberries improved post-prandial glucose concentration due to high fat and inflammation and oxidation in diabetic individuals.	[74]
Helicobacter pylori suppression	Non-dialyzable substances from cranberry obstruct the sialic acid-specific adhesion of *H. pylori* to human gastric mucus and to erythrocytes.	[75,76,77,78,79,80]
Cancers	*Prostate*-Cranberry PACs reduced matrix metalloproteinases (MMP) activity in prostate cancer cells via stimulating and hindering specific MMP regulators, and by disrupting either the phosphorylation status and/or expression of MAP kinase, PI-3 kinase, and NF-κB and AP-1 pathway proteins. *Bladder*-Isorhamnetin and quercetin 3-O-glucoside, the active forms of quercetin may be responsible in prevention of bladder cancer in vivo and diets high in cranberries for the prevention of bladder carcinoma. *Breast*-Cranberry phytochemical may potentially suppress the spread of human breast cancer MCF-7 cells, which is partly due to both the beginning of apoptosis and the G1 phase arrest. *Lung*-PACs in cranberry can modify gene expression, stimulate apoptosis and induce the cell cycle of human NCI-H460 lung cancer cells.	[81,82,83]
Rheumatoid arthritis	Quercetin, a flavonoid present in cranberry, is a powerful suppressor of the nuclear factor (NF)-ĸB-pathway. It also impedes the activities of cyclooxygenase and lipoxygenase, enzymes released after the stimulation of arachidonic acid, which is the initiator of an inflammatory response. Resveratrol, a polyphenol in cranberry, also has been shown to reduce inflammatory genes expression important for cardiovascular disease by regulating the NF-ĸB and JAK STAT3 pathways in cells.	[84]
Tooth decay and periodontitis	Polyphenols in cranberry serve as dental anticaries agents by impeding the production of organic acids and the formation of biofilms by cariogenic bacteria. Additionally, they may reduce inflammation as well as the production and activity of proteolytic enzymes destroying the extracellular matrix in periodontal disease. These polyphenols also interfere with other activities such as formation of biofilm and adhesion of *Porphyromonas gingivalis*, the main disease-causing agent in chronic periodontitis.	[85,86,87,88]
Infectious	PAC in cranberries block adhesion to and biofilm formation on target tissues of pathogens	[89]
Kidney	Cranberries enriched with PACs can alleviate the complications associated with chronic kidney disease such as oxidative stress, inflammation and gut dysbiosis	[90]
Intestinal microbiota	The rich cranberry content of polyphenols, phenolic acids, isoprenoids and oligosaccharides performing in the gastrointestinal tract may reduce reactive oxygen species, control pathways of inflammation, attach to carbohydrates and proteins on surfaces of bacteria, employ prebiotic effects, and change the transmission of signals between intestinal epithelial cells and the gut microbiota.	[91]
Flu virus	High molecular weight substances (NDM) in cranberry inhibited Influenza virus A subtypes (H1N1 and H3N2) and the B type, which was shown by the cytopathic effect on Madine-Darby canine kidney (MDCK) cells and the lack of hemagglutination of red blood cells activity in infected cells.	[92,93]
Microbial	Cranberry phenolic extracts impeded the growth of human pathogenic bacteria: *Escherichia coli*, *Salmonella typhimurium*, *Enterococcus faecalis*, *Listeria monocytogenes*, *Staphylococcus aureus*, and *Bacillus subtilis* in different mechanisms.	[94,95,96,97]
